# Assessing the Readiness of Rural Public Librarians to Implement Public Health Programs

**DOI:** 10.1007/s10900-024-01402-0

**Published:** 2024-09-06

**Authors:** Noah Lenstra, Heather Franklin, Nathan F. Dieckmann, Elena Andreyeva, Jay Maddock, Rebecca A. Seguin-Fowler, Jim Winkle, Cynthia K. Perry

**Affiliations:** 1 Department of Information, Library, and Research Sciences, University of North Carolina at Greensboro, Greensboro, USA; 2 Oregon Health & Science University School of Nursing, Portland, OR, USA; 3 Texas A&M School of Public Health, College Station, TX, USA; 4 Texas A&M Institute for Advancing Health through Agriculture, College Station, TX, USA

**Keywords:** Rural health, Health promotion, Community health workforce

## Abstract

Although health promotion is not the primary function of public libraries, it is well documented that many libraries engage in health promotion activities, even when resources are constrained. Less understood is the readiness of the public library workforce, particularly in rural communities, to implement evidence-based health promotion programs. This study uses a modified version of the Competency Assessment for Tier 2 Public Health Professionals to assess the readiness of a small sample (*n* = 21) of Oregon rural library managers to implement evidence-based health initiatives. Results show that outside of communication skills, most rural library workers do not consider themselves to have proficiency in core health promotion competencies. Although some slight differences were found among librarians based on socio-demographic factors, those differences were not statistically significant. Implications include the need for strengthened support to build the capacity for rural public library workers who are interested in delivering evidence-based health promotion programs.

## Background

The past decade has seen increased interest in how and why public libraries engage in health promotion activities [1–3] Within the field of public health, public libraries are increasingly seen as organizations which, alongside others such as faith-based organizations, community-based organizations, and public housing communities, can play key roles as community-level resources to advance population health [4, 5].

Rural residents are at a disadvantage in terms of access to health promotion activities, including access to the social spaces that in part determine health outcomes. A recent study of rural poverty concluded that the absence of social infrastructure, or places where people can engage in a range of activities together [6], may be one cause of negative health outcomes in rural America. Given this reality, rural libraries, especially if better supported, may become key social infrastructure in rural America. More than half of all U.S. public libraries serve populations fewer than 10,000, and the average rural American lives about 4.9 miles from a library. Based on the most recent data, the 30 million Americans served by the nation’s 4,000 rural library systems visit these libraries 117 million times annually [7]. Rural libraries have been studied as health promotion partners, finding them to be often dynamic, socially responsive institutions and are becoming community hubs that offer a multitude of programs and services, including health promotion activities [8–11]. Many rural libraries engage in health promotion activities, even when resources are constrained [12].

Implementing public health programs in rural United States of America (US) libraries requires understanding of how these governmental institutions function. Each state in the US determines its own minimum standards for public library staffing. In Oregon, the state where this study took place, the only staffing requirement is that a public library that serves a population of 2,000 or more dedicate at least 0.50 full-time equivalent (FTE) paid staff exclusively to library functions. In 2022, across the state of Oregon, the total paid staff of public libraries was more than 1,900 FTEs, 23% of whom had a master’s degree in library & information science [13]. Like public libraries in other US states, Oregon’s public libraries are at risk of being underfunded, defunded, and in some cases even privatized [14]. Nevertheless, Oregon’s public libraries continue to develop innovative partnerships and programs to support their communities [15].

At the national level, the field of public librarianship has adapted over the last forty years from one primarily focused on providing physical access to materials to one primarily focused on supporting access to lifelong learning and literacy; digital and social inclusion; and community gathering places [16–19]. These changes can be found within public health research on how and why public libraries engage in health promotion activities. For instance, Maryland public library staff see health promotion activities fitting within the mission of the library to support (1) the education of the public, (2) access to reliable information, and (3) social equity and inclusion [4].

Although there have been national calls to increase public library staff competencies for the support of consumer health literacy and other health promotion activities [20–22], it is unknown to what extent these competencies are embedded within the workforce. The Robert Wood Johnson Foundation and the Public Library Association invested in training librarians to support the roll-out of the US Affordable Care Act [23], but without an evaluation of the competencies that resulted from this training. Furthermore, these consumer health competencies developed for librarians tend to focus on helping librarians support individuals through one-on-one interactions focused on gathering information related to medical diagnosis, or finding health insurance. These competencies do not focus on how to administer community-level interventions. No study has examined the preparedness of public libraries to implement evidence-based health promotion programs with groups of people in their local communities.

Past studies of core competencies for the public health workforce have found these metrics can help guide future training for individuals engaged in leading health promotion activities [24]. Notably, some research has endeavored to assess the public health competencies not only of trained public health professionals, but also of lay individuals engaged in health promotion leadership. An evaluation of how members of cancer coalitions communicate about public health, concluded most have limited proficiency prior to a training intervention [25]. Assessments of public health competencies tend to be related to internal strategic planning exercises. Nevertheless, some results have been published. A study of public health program managers in South Carolina – most of whom lack formal public health education – found workforce to be strongest in communication and cultural competency, and weakest in financial planning and assessment [26]. A similar study in Montana found public health program supervisors to be most competent in communication, and weakest in systems thinking [27]. The purpose of this study is to assess to what extent participating rural library managers perceive themselves to be proficient in competencies necessary for local leadership of evidence-based health programs.

## Methods

As part of a National Institutes of Health (NIH) funded cluster randomized trial testing the effectiveness of walking groups or walking groups plus civic engagement for walkability in rural communities via rural libraries [28], rural library managers across Oregon were recruited to serve as local leaders and hosts of the intervention. Library staff were recruited to participate in this project using an iterative process that involved emails sent through state library organizations, direct phone calls, site visits, and snowball sampling.

### Measures

As part of the study, library managers were asked to complete 3 surveys over a 13-month time period. The first survey asked managers to complete a self-assessment of competencies associated with public health professionals who have supervisory responsibilities for public health interventions or initiatives, based on a version of the 2014 Competency Assessment for Tier 2 Public Health Professionals [29]. The assessment captured competencies in seven domains, including Analytic and Assessment Policy, Development and Program Management, Communication Skills, Cultural Competency, Community Dimensions of Practice, Leadership and Systems Thinking, and Financial Planning and Management. The domain Public Health Sciences Skills was not included since it was not relevant to implementing evidence-based programs. The assessment required managers to respond to their perceived level of competency in individual skills within each domain using Likert Scale responses (1-no or very little knowledge of the skill; 2-heard of the skill but had limited knowledge of and had limited ability to apply it; 3-comfortable in their ability to apply the skill; 4- expert in the skill and could teach it to others). The survey contained 45 items.

In addition, the Summer 2023 survey collected data on managers’ educational and professional backgrounds, such as the length of time they had worked at their libraries, and in libraries more generally. The Spring 2024 survey collected data on managers’ prior experiences administering projects that involved the recruitment and retention of members of the project, as well as their demographic backgrounds.

### Sample

After recruitment into the project, 21 rural library managers (henceforth referred to as managers), located throughout all population centers of the state, completed survey #1. Due to staffing transitions in these libraries, 20 completed survey #2 and 17 completed survey #3.

### Analytic Approach

Responses to the Competency Assessment items were collapsed into the categories of “not proficient” (responses 1 and 2) or “proficient” (responses 3 and 4) and total proficiency score was calculated for each participant. If one were proficient in all seven competencies, one would score a “7,” if one was not proficient in any competency, one would score a “0.” Standard statistics (e.g., proportions and means/standard deviations) were used to describe the sample, and t-tests were used to compare perceived proficiency across sociodemographic and professional characteristics.

## Results

### Socio-Demographic and Professional Variables

The sample was primarily white and female. The average age reported was 49. Most lived in the community served by their library (Table 1). Over half of the managers (61.9%) reported working in the library profession for 11 years or more, and nearly a quarter (23.8%) reported working at their *current* library for 11 years or more. Nine (42.9%) had attained a master’s degree in library & information science (the standard professional degree for the field), while most (71.4%) were members of a professional association of librarians. There was high variation in terms of time available annually for professional development, with 4 (19.0%) having 10 or less hours, 4 (19.0%) having 11–20 h, 5 (23.8%) having 21–40, 3 (14.3%) having 41–60, and 2 (9.5%) having 61 h or more of time annually to dedicate to professional development and continuing education.

In terms of prior professional experiences related to being a local leader of an evidence-based health intervention, 9 (42.9%) reported offering a program series for adults, beyond book clubs, while slightly less (8, 38.1%) reported no such experiences. Examples of program series for adults offered included a speaker series on health and wellness topics, and a series of classes on gardening. Fewer (5, 23.8%) reported experiences that explicitly required them to recruit adults to participate in initiatives, with other library programs and classes being drop-in. Examples of efforts for which managers recruited adults other than for programs included recruiting library volunteers, recruiting adults to serve on the library board, and recruiting adults to participate in community conversations held at the library.

### Proficiency in Public Health Competencies

Managers who completed this survey reported themselves to be most proficient in communication, the only competency where more than 50% reported proficiency (Table 2). Managers reported themselves least proficient in analytical/assessment and in community dimensions of practice skills. Overall, less than one-third of managers reported being proficient in four or more competencies. In total, 7 managers scored “0,” meaning no proficiency in any competencies; 8 reported proficiency in 1–3 competencies; 4 reported proficiency in 4 to 6 competencies; and 2 reported proficiency in all 7 competencies.

### Comparing Proficiencies to Socio-Demographic Variables

Analysis of total proficiency score by professional and demographic characteristics revealed no statistically significant relationships between sociodemographic/professional characteristics and proficiency score. A number of statistical tests were run to attempt to discern relationships, and these tests were inconclusive in terms of patterns.

## Discussion

In general, some library managers see themselves as having some proficiency with some of the skills recognized as essential for implementing local health promotion initiatives. Managers reported feeling most comfortable with communication skills, and least comfortable with community dimensions of practice skills, such as identifying stakeholders and maintaining collaborations with them, and analytical/assessment skills involved in program evaluation. These results partially align with other studies of managers of health promotion activities, who were also found to be most competent in communication skills while struggling in other domains of practice [25–27].

Comparing aggregate proficiency to a variety of socio-demographic variables, furthermore, does not reveal any that could explain the differences between library managers that have competencies, and those that do not. Additional research is needed to better understand why some rural library managers have proficiency in the skills needed for public health intervention management, while others do not.

Echoing past studies of competencies perceived as valuable or necessary to contemporary public librarianship [16–18], this study found that competencies associated with communication skills and cultural competency were most present among rural library managers. This study also adds to the evidence-based research showing that many libraries have an interest in health promotion activities, even when resources are constrained [3/10]. Even rural library managers who had no dedicated time for professional development, and who lack competencies related to health promotion, chose to add this project to their administrative duties. The willingness of rural library managers to become involved in public health despite these constraints speaks to the need for greater support for this workforce in the public health ecosystem, alongside other non-traditional public health workers.

Future work should focus on helping rural libraries build competencies through structured opportunities to work collaboratively with others to improve public health. One promising strategy could be to connect rural librarians to community health coalitions, where mutual learning and resource sharing among members may occur. For instance, McGladrey et al. [30] describe recruiting a representative from a public library in rural Kentucky to a coalition that in turn set a common agenda for improving physical activity. The development of skills related to leading health promotion activities in the Oregon rural library workforce who participated in this study, may have spill-over effects, increasing the capacity of this vital anchor institution to operate in an efficient, effective way. Alternatively, this study also suggests that public health organizations looking to rural libraries to implement programs offer the services of a statistician, or otherwise avoid requiring that the library perform analysis or assessment. Technical assistance could also be provided to help overcome limitations in this workforce.

### Limitations

This study is based on a sample of 21 rural library managers in one state. Additional research is needed to further expand our understanding of the readiness of public libraries, and rural libraries in particular, to participate in and help lead public health interventions. This article is limited by its small sample size, which is not a random selection of rural libraries in Oregon or across the United States. Nevertheless, given the fact that to participate in this study rural library managers had to self-identify as having the capacity to lead the local implementation of a state-wide health promotion initiative, it would be reasonable to conclude that they self-identify as being more proficient than other rural library managers in Oregon. In other words, it would be reasonable to conclude that rural libraries that chose not to participate in this study identify themselves as having even less proficiency, and thus may require even more support to be able to participate in and lead evidence-based health promotion initiatives in the future. Finally, all proficiency data is self-reported. Results on objective measures of proficiency may differ.

## Conclusions

This study sought to assess the preparedness of rural public library managers to participate in and to help lead health interventions. Although few reported expertise in all the skills or competencies, most respondents reported comfort and familiarity with communications and cultural competence skills, and less comfort and less familiarity with skills associated with evaluation, assessment, program planning, and developing and participating in communities of practice. Future work could focus on building those competencies while also extending our understanding of rural libraries as part of public health ecosystems across rural America.

## Figures and Tables

**Table 1:** Sociodemographic and professional variables

	Overall (*n* = 21)

**Years worked at your current library**	
Less than five years	5 (23.78%)
5–10 years	9 (42.9%)
11–15 years	1 (4.8%)
16–20 years	3 (14.3%)
20 years or more	1 (4.8%)
Missing	2 (9.5%)
**Years worked in the library profession**	
Less than five years	2 (9.5%)
5–10 years	4 (19.0%)
11–15 years	4 (19.0%)
16–20 years	4 (19.0%)
20 years or more	5 (23.8%)
Missing	2 (9.5%)
**Highest level of education completed**	
Master’s degree in library & information science or related	9 (42.9%)
Other Master’s or post baccalaureate degree	1 (4.8%)
Undergraduate degree	4 (19.0%)
Associates degree	2 (9.5%)
High School	3 (14.3%)
Missing	2 (9.5%)
**Member of professional associations of librarians (e.g. Oregon Library Association)**	
No	4 (19.0%)
Yes	15 (71.4%)
Missing	2 (9.5%)
**Time available for professional development on annual basis**	
10 or less hours	4 (19.0%)
11–20h	4 (19.0%)
21–40h	5 (23.8%)
41–60h	3 (14.3%)
61 + hours	2 (9.5%)
Missing	3 (14.3%)
**Live in the community served by your library**	
No	4 (19.0%)
Yes	16 (76.2%)
Missing	1 (4.8%)
**Race or ethnic background**	
White	15 (71.4%)
Asian	1 (4.8%)
Latino	1 (4.8%)
Missing	4 (19.0%)
**Gender**	
Woman	13 (61.9%)
Man	4 (19.0%)
Missing	4 (19.0%)
**Age**	
30–39	4 (19.0%)
40–49	3 (14.3%)
50–59	7 (33.3%)
60–69	3 (14.3%)
Missing	4 (19.0%)
**Library has offered a program series for adults, beyond book clubs**	
Yes	9 (42.9%)
No	8 (38.1%)
Missing	4 (19.0%)
**Maximum length of program series offered for adults at library**	
4 weeks	2 (9.5%)
12 weeks	1 (4.8%)
Continuously (meet on an ongoing basis)	6 (28.6%)
No program series for adults	8 (38.1%)
Missing	4 (19.0%)
**Librarian has had to recruit adults to programs in the past**	
Yes	5 (23.8%)
No	12 (57.1%)
Missing	4 (19.0%)
**All work for this project completed during work hours**	
Yes	16 (76.2%)
No	1 (4.8%)
Missing	4 (19.0%)

**Table 2:** Proportion of managers (*n* = 21) proficient in competencies

	Not proficient		Proficient	
	
Competency	No proficiency	Limited Proficiency	Some Proficiency	Total Proficiency

Communication	0 (0.0%)	10 (47.6%)	9 (42.9%)	2 (9.5%)
Cultural Competency	0 (0.0%)	12 (57.1%)	8 (38.1%)	1 (4.8%)
Leadership and systems thinking	3 (14.3%)	11 (52.4%)	6 (28.6%)	1 (4.8%)
Policy Development and Program Planning	2 (9.5%)	14 (66.7%)	5 (23.8%)	0 (0.0%)
Financial planning and management	3 (14.3%)	13 (61.9%)	5 (23.8%)	0 (0.0%)
Analytical/Assessment	3 (14.3%)	14 (66.7%)	4 (19.0%)	0 (0.0%)
Community Dimensions of Practice	4 (19.0%)	13 (61.9%)	4 (19.0%)	0 (0.0%)
